# Longitudinal change in speech classification between 4 and 10 years in children with cerebral palsy

**DOI:** 10.1111/dmcn.15198

**Published:** 2022-03-09

**Authors:** Helen L. Long, Tristan J. Mahr, Phoebe Natzke, Paul J. Rathouz, Katherine C. Hustad

**Affiliations:** 1 Waisman Center, University of Wisconsin–Madison, Madison, Wisconsin, USA; 2 Dell Medical School, Department of Population Health, University of Texas at Austin, Austin, Texas, USA; 3 Department of Communication Sciences and Disorders, University of Wisconsin–Madison, Madison, Wisconsin, USA

## Abstract

**Aim:**

To examine speech impairment severity classification over time in a longitudinal cohort of children with cerebral palsy (CP).

**Method:**

A total of 101 children (58 males, 43 females) between the ages of 4 and 10 years with CP participated in this longitudinal study. Speech severity was rated using the Viking Speech Scale (VSS), a four-level classification rating scale, at 4, 6, 8, and 10 years (age 4 years: mean = 52 months [3 SD]; age 6 years: mean = 75 months [2 SD]; age 8 years: mean = 100 months [4 SD]; age 10 years: mean = 125 months [5 SD]). We used Bayesian mixed-effects ordinal logistic regression to model (1) the extent to which speech severity changed over time and (2) patterns of change across age groups and classification rating group levels.

**Results:**

VSS ratings decreased (speech severity became less severe) between 4 and 10 years of age. Children who were first classified in VSS levels I, II, or III at age 4 years had a high probability of staying at, or improving to, VSS level I by 10 years. Children who were first classified in VSS level IV at 4 years had a high probability of remaining in VSS level IV at 10 years.

**Interpretation:**

Early speech performance is highly predictive of later childhood speech abilities. Children with any level of speech impairment at age 4 years should be receiving speech therapy. Those with more severe speech impairments should be introduced to augmentative and alternative communication as soon as possible.

Cerebral palsy (CP) is one of the most common motor disorders in childhood.^1^ Speech impairments may affect over 80% of children with CP.^2^ In addition, language, cognitive, and functional communication limitations may be present. Communication profiles among individuals with CP are diverse and heterogeneous.^3–5^

Classification tools have gained widespread use as a means of reducing heterogeneity, establishing a common vocabulary for describing similar features among individuals, and for epidemiological research. Standard clinical practice involves application of the Gross Motor Function Classification System (GMFCS)^6^ and the Manual Ability Classification System (MACS)^7^ to describe functional motor abilities in children with CP. These measures are well validated^8–13^ and widely used internationally. Similar classification systems have been developed for communication. For example, the Functional Communication Classification System^14^ and the Communication Function Classification System^15^ are five-level ordinal rating systems that parallel the GMFCS and MACS and are used to measure global communicative function abilities in children with CP.

The Viking Speech Scale (VSS)^16^ is another classification tool that focuses specifically on speech in individuals with CP. The VSS was designed to describe speech motor involvement severity, with particular reference to intelligibility and the functional consequences of intelligibility limitations in children over 4 years of age. A key difference between the VSS and other communication-related classification tools is that it exclusively considers speech abilities, whereas other tools consider communication more broadly and include the use of augmentative and alternative communication (AAC) supports. The VSS is increasingly used in research and clinical contexts.^17–19^ Like other classification systems, the VSS requires raters to use subjective judgment to assign individual speakers to one of four different ordinal severity categories. The VSS has shown good-to-excellent interrater agreement among clinicians.^20^ Pennington and Hustad^21^ found strong relationships between single-word and connected speech intelligibility measures with VSS scores in children in the USA and UK, suggesting that VSS ratings adequately reflect a speaker’s overall ability to be understood.

The available research offers convincing evidence on the reliability and validity of the VSS for classifying speech impairment severity in CP.^20–24^ However, studies have not examined the impact of speech growth and development on VSS ratings. Thus, we do not know whether children should be expected to show improved VSS ratings as a function of development over time or whether VSS ratings should be expected to remain static over time. If VSS ratings indeed change over time, the extent to which early ratings may predict later outcomes could provide important prognostic information that informs the direction of interventions. For example, if a child is classified in VSS level IV (the most severe) at an early age and if that classification has a high probability of staying the same over time, it suggests that early comprehensive AAC interventions would likely be an important treatment direction. If, however, a child is classified in VSS level IV at an early age and if that classification has a high probability of improving over time to VSS level III or even level II, it suggests that therapy directions should include a focus on both speech production and AAC to enhance multimodal communication.

Classification of gross and fine motor skills, as measured by the GMFCS and MACS, at ages older than 2 years tend to be stable over time^9,25,26^ with some evidence of downward GMFCS trends, that is, decreasing severity observed across mid-classification levels.^11^ However, speech development differs from other motor development domains. Notably, recent research suggested that speech intelligibility is still developing for many typically developing children beyond 9 years of age.^27,28^ Similarly, studies of speech intelligibility growth in children with CP suggested a very protracted time course,^29^ with periods of most rapid growth occurring more than a year later than expected for typical peers.^30,31^ Given this growth lag and the protracted course of development among children with and without speech motor impairment, it is important to understand how developmental change translates to ordinal rating scale classification on the VSS and the extent to which early ratings are predictive of later ratings. Toward this end, we sought to address the following research questions in the present study: (1) To what extent do VSS levels change with development in a longitudinal cohort of children with CP between 4 and 10 years of age? (2) How well do VSS levels at 4 years predict VSS levels at 10 years?

We predicted that speech impairment in children with CP would become less severe over time, as measured by the VSS. This prediction is based on previous studies demonstrating that intelligibility increases with development; therefore, the perception of severity of speech involvement should decrease with development, given that severity is partially related to intelligibility. We expected that initial VSS levels at 4 years would be predictive of later ratings, with children showing lower severity ratings over time, particularly for those children in the ‘moderate’ range (i.e. VSS levels II and III). We also expected that there would be subsets of children who did not show change over time and that those children would primarily fall on the poles of the ordinal scale, such that children classified in VSS level I (the least severe) would maintain this rating at later ages and children classified in VSS level IV (the most severe) would be more likely to maintain this rating at later ages.

## METHOD

This study was approved by the institutional review board for social and behavioral sciences at the University of Wisconsin–Madison (no. 2018-0580). Written informed consent or assent was provided on behalf of or by all participants.

### Participants

A total of 101 children with CP (58 males, 43 females) and their families consented to participate in this study. Children were part of a larger longitudinal project (*n* = 139) examining communication development in children with CP. All participants had a primary medical diagnosis of CP and hearing within normal limits as determined by a formal audiological evaluation or distortion product otoacoustic emission screening. Children with co-diagnoses were not excluded from this study. From the full sample of 139, we sought children who had completed at least two sessions across four possible age groups: between the ages of 4 and 5 years (age 4 years); between the ages of 6 and 7 years (age 6 years); between the ages of 8 and 9 years (age 8 years); and between the ages of 10 and 11 years (age 10 years) (Table 1). Individual children were only allowed to contribute to one visit per age group.

In the final analysis, 101 children with CP were included. Collectively, they contributed 328 total visits, yielding a mean of 3.25 (SD = 0.82) and median of 3 longitudinal visits per child. All children were from homes where American English was the primary language. Children were born in the United States between 2000 and 2009. Summary statistics of the number of children at each age and demographic information are provided in Tables 1 and 2 respectively.

### Measures and procedures

#### VSS ratings and stimuli

Speech impairment severity was classified for children at each age studied into one of four ordinal severity levels of the VSS.^16^ The VSS classification material describe the levels as follows: children in level I have speech that is not affected by a motor disorder; children in level II have imprecise speech that is usually understandable to unfamiliar listeners; children in level III have speech that is unclear and not usually understandable to unfamiliar listeners out of context; children in level IV have no understandable speech.

Four judges made ratings of the VSS levels. Judges were research assistants who had an academic background in speech language pathology but did not have prior familiarity with the specific children in the study. Judges were given a brief introduction to the use of the VSS rating system and its purpose; they were provided a full copy of the VSS^16^ as reference while rating the children. This document describes each level of classification and perceptual characteristics that can be used to differentiate each level.

Each judge was assigned to classify children’s speech impairment severity at a single age group: ages 4, 6, 8, or 10 years, with one judge rating all 4-year-old children, a different judge rating all 6-year-old children, and so on. Judges used two types of recordings collected in a laboratory setting to calculate the VSS ratings: (1) approximate 10-minute structured video recordings of a parent–child interaction; and (2) audio recordings of the child’s repetition of a set of speech stimuli from the Test of Children’s Speech Plus^32^ from 1 to 7 words in length. Judges used headphones while listening to audio and video samples. They were encouraged to watch and listen to the samples as many times as they needed before making their classifications. Judges made their ratings independently and did not consult one another during the process. They completed their primary and reliability ratings within a 2-week period. All judges were blinded to the ratings made by other judges of children at different ages.

### Reliability

Intrarater reliability was assessed by having the four judges reassess a subset (15–26%) of children. These reassessments were made, on average, 2 weeks after the initial ratings. Cohen’s kappa with squared weights showed strong-to-perfect intrarater agreement for the four raters (*κ* = 0.92, *κ* = 0.81, *κ* = 1.00, and *κ* = 0.76).

Interrater reliability probes were completed by having a second rater score a subset of samples (15–43%) for each of the four ages, with one judge scoring all 4-year-old reliability samples, another scoring all 6-year-old reliability samples, and so on. Cohen’s kappa with squared weights likewise showed strong-to-near-perfect agreement (*κ* = 0.82, *κ* = 0.80, *κ* = 0.95, and *κ* = 0.96 for ratings at ages, 4, 6, 8, and 10 years respectively). Kappa statistics were computed using the irr R package (version 0.84.1).^33^

### Statistical analysis

We used Bayesian mixed-effects ordinal logistic regression models^34^ to estimate how the speech ratings changed with age in this sample. The approach—also called a proportional odds model—assumes that there is a latent continuous speech severity variable and that the ordinal VSS levels divide up regions of that latent speech variable. The model estimates the thresholds separating the rating levels and the probability of each rating level. We used the Bayesian approach for computational feasibility (it can estimate this kind of model) and for uncertainty propagation (it can estimate the uncertainty in model-derived quantities, such as marginal means). We used the modeling software’s default flat and weakly informative priors. Appendix S1 provides a detailed description of the model.

The models for both research questions included age as a predictor. This age effect estimated how rating thresholds and probabilities changed with age for an average child. The mixed effects account for the longitudinal structure of the data and allow for incomplete data (children with ratings at just two or three ages) when estimating how ratings change with age. The models included by-child random intercepts, shifting the thresholds for each child, and by-child random age slopes for age, giving each child their own age effect.

Appendix S1 provides the modeling syntax used to fit models and compute marginal rating probabilities from a fitted model. These marginal means allowed us to estimate the expected ratings that account for child-level variability in estimating statistical parameter uncertainty. For the first research question, the sole predictor in the model was age. For the second research question, we extended the model’s predictor set to include initial (age 4 years) VSS level as a categorical predictor variable and an age-by-initial rating interaction. For this interaction, we coded the initial rating as an integer so that the age effect (i.e. age slope) would change linearly with the initial rating level.

We estimated the models using Stan (version 2.27.0)^35^ via the brms (version 2.16.1)^36^ and tidybayes packages (version 3.0.1)^37^ in R (version 4.1.2).^38^ The model used the default flat priors for the fixed-effects parameters and weakly informative priors for the random-effects parameters and passed all sampling diagnostics.

## RESULTS

### Research question 1: To what extent do VSS levels change with development in children with CP between ages 4 and 10 years?

**FIGURE 1:**
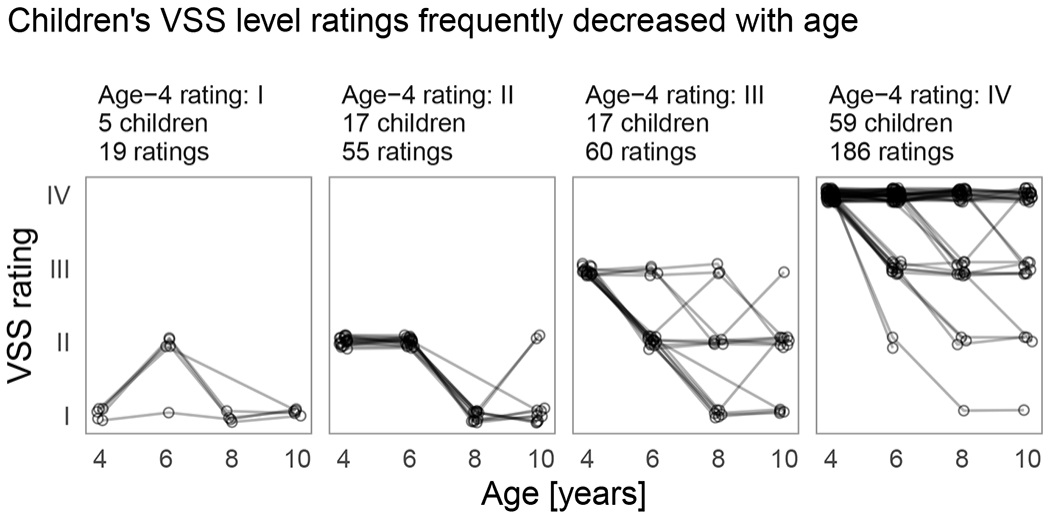
Children’s Viking Speech Scale (VSS) level ratings frequently decreased with age. Observed VSS levels by initial (age 4 years) rating. Each line connects the VSS levels (points) for a single child. Data from three children without an age 4 years VSS level are not included in this figure

We first describe the observed data and then report the model results. Figure 1 presents the results by initial VSS level with one line per child. VSS levels were more likely to decrease (become less severe) with age or remain the same rather than increase with age. We can describe this age effect by counting how many times the lines in Figure 1 change direction (a within-child age-to-age change in ratings). Out of 227 possible age-to-age changes in VSS levels, there were 65 decreases in VSS level, compared to 10 increases in VSS level, and 152 instances where the VSS levels did not change between ages. The allocation of ratings also shows how VSS levels decreased with age. Fifty-six children had the same VSS levels at ages 4 and 10 years. The allocation of ratings across VSS levels I, II, III, and IV at age 4 years was 5, 10, 13, and 28 children compared to 18, 13, 8, and 17 children at age 10 years. Notably, over the 6-year age range of the study, the least severe rating (VSS level I) changed from being the least numerous to the most numerous in the sample.

The proportional odds model’s estimated age effect odds ratio (OR) was 5.7 (with a 95% credible interval [CI] 3.2–11.6) for each 2-year increase in age. (A 95% credible interval is the Bayesian analogue of a 95% confidence interval in classical inference for describing uncertainty around a point estimate.) That is, the odds for a ‘low’ (less severe) versus ‘high’ (more severe) rating of VSS level was 5.7 times higher for an average 6-year-old child compared to an average 4-year-old child. The result can be converted to estimated probabilities. For example, the estimated probability of a VSS level of III, II, or I for an average child was 0.27 (95% CI 0.07–0.59) at age 4 years and 0.67 (95% CI 0.31–0.90) at age 6 years.

**FIGURE 2:**
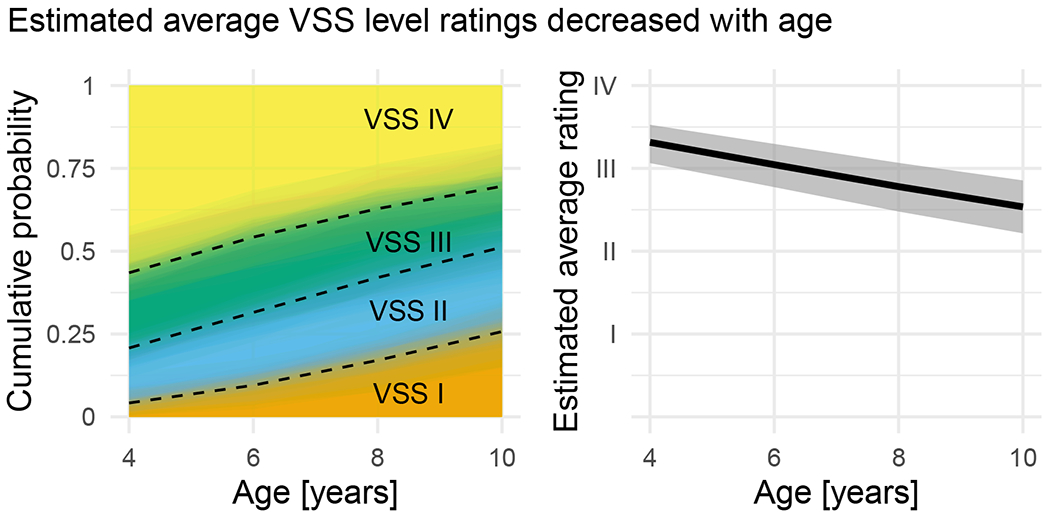
Estimated average Viking Speech Scale (VSS) level ratings decreased with age. (a) Estimated VSS level probabilities by age. The average probability of the VSS levels changed with age such that less severe (lower) levels were more likely at older ages. The dashed lines show the posterior medians of the Bayesian estimates of the cumulative level probabilities. The filled areas depict 100 posterior samples of the level probabilities, so the color gradations around the dashed lines depict the uncertainty in cumulative probabilities. (b) Estimated posterior median average VSS levels by age and 95% credible interval. By multiplying each level’s numerical value (1–4) by its probability (a) and summing these weighted values, we can estimate the average VSS level

By averaging over the fitted distribution of children (instead of describing an average child), we estimated how the marginal probabilities changed with age. Figure 2 visualizes the change in marginal probabilities over time. For example, at age 4 years, VSS levels had the following fitted probabilities: *p*(1) = 0.04 (0.01–0.11), *p*(2) = 0.16 (0.10–0.24), *p*(3) = 0.22 (0.15–0.31), *p*(4) = 0.57 (0.44–0.68). At age 10 years, the probabilities had shifted so that less severe VSS levels were more likely: *p*(1) = 0.26 (0.16–0.37), *p*(2) = 0.25 (0.17–0.34), *p*(3) = 0.18 (0.12–0.26), *p*(4) = 0.30 (0.19–0.44). Thresholds between VSS levels (the dashed lines in Figure 2) all increased with age. The smallest change in marginal threshold was for VSS levels I, II, and III versus level IV between the ages of 8 and 10 years, OR = 1.3 (95% CI 1.1–1.7), and the largest change was for VSS level I versus levels II, III, and IV between the ages of 4 and 6 years, OR = 2.4 (95% CI 1.5–6.8).

Estimated average VSS levels (the weighted average of rating values times the marginal rating probabilities [Figure 2]) provide another description of this age effect. At age 4 years, the expected VSS level was 3.3 (95% CI 3.1–3.5). Expected VSS levels decreased with each age step: 3.0 (95% CI 2.8–3.3) at age 6 years; 2.8 (95% CI 2.5–3.1) at age 8 years; and 2.5 (95% CI 2.2–2.9) at age 10 years. The difference in expected VSS levels between the ages of 10 and 4 years was −0.8 (−1.0 to −0.6), indicating that VSS levels decreased with development and the magnitude of this decrease was somewhere between a half and a full VSS level.

### Research question 2: How well do VSS levels at age 4 years predict VSS levels at age 10 years?

Table 3 provides the age 10 years VSS levels by initial age 4 years VSS levels as both observed data and model estimates. Note that we are interested in conditional probabilities such as the probability of receiving a rating of I at age 10 years *given* an initial age 4 years rating of I. We use the notation *p*(I | initial I) for these probabilities. For a child with an initial VSS level of I or II at age 4 years, the most likely outcome at age 10 years was VSS level I. All five children who were initially rated in VSS level I remained in level I and the model estimated the probability of VSS level I at age 10 years to be nearly 1 (probability of an age 10 years I rating *given* an age 4 years I rating: *p*[I | initial I] = 0.98, 95% CI 0.86–1.0). For children initially rated in VSS level II, 8 out of 10 children were rated in VSS level I at age 10 years and this supports the model-estimated probability of a VSS level I of 0.87 (95% CI 0.69–0.97).

For a child in VSS level III at age 4 years, the most likely age 10 years VSS level was I or II, each with similar probabilities. For the 13 children initially rated in VSS level III, four were rated in VSS level I and eight were rated in VSS level II at age 10 years. Thus, VSS level II was the most likely observed outcome. Of the four children who did not return at age 10 years, two were classified in VSS level I at age 8 years. The model estimates, which incorporated this partial information, estimated a higher probability of VSS level I (*p*[I | initial III] = 0.48, 95% CI 0.30–0.67, than VSS level II, *p*[II | initial III] = 0.39, 95% CI 0.24–0.55). However, these are similar probabilities and their overlapping credible intervals indicate some uncertainty about whether VSS level I or II was the most likely outcome. In 28% of posterior samples, the estimate of *p*(II | initial III) was greater than the estimate of *p*(I | initial III).

For an initial VSS level of II or III, we expect age 10 years to be at least one level lower than the age 4 years level. However, for a child in an initial VSS level of IV, the most likely age 10 years outcome was VSS level IV. For our observed ratings, about half of the children who were initially in VSS level IV did not return at age 10 years (31 out of 59). Of the 28 children who returned at age 10 years, 17 were in VSS level IV and seven were in VSS level III. For children with partial data—visits at ages 4, 6, and 8 years but not 10 years—there was a similar allocation at age 8 years: 10 children were in VSS level IV, three were in VSS level III, and one was in VSS level II. The model estimates assigned the highest probability to VSS level IV (*p*[IV | initial IV] = 0.62, 95% CI 0.45–0.77), followed by VSS level III (*p*[III | initial IV] = 0.23, 95% CI 0.12–0.36). Compared to the other initial ratings, VSS level IV at age 4 years was highly predictive of age 10 years outcomes.

## DISCUSSION

In this study, we examined longitudinal change in speech impairment severity as measured by the VSS for 101 children with CP between the ages of 4 and 10 years. We examined this age range because it is a key time frame when children have been shown to make important gains in speech intelligibility development.^28–30^ Our main objectives were to establish whether speech performance growth would be reflected in lower VSS levels over time across children with CP. We also sought to examine the extent to which VSS levels observed at age 4 years were differentially predictive of VSS levels at age 10 years. There were two key findings from this study. First, children with CP demonstrated decreasing trends in VSS levels over time between the ages of 4 and 10 years (i.e. became less severe). Second, early VSS levels were highly predictive of outcomes, with each of the four VSS levels at age 4 years yielding different predicted outcomes at age 10 years. These findings are discussed in detail in the next sections.

### VSS levels improve (become less severe) with age in children with CP

Children make important refinements in their speech production ability between the ages of 4 and 10 years. Recent large-scale work on intelligibility demonstrated this, indicating that the intelligibility of an average typically developing child at 4 years for connected speech is 78%, while intelligibility for an average 10-year-old child is 98%.^28^ Children with CP can make gains of a similar or even larger magnitude, even in the presence of severe dysarthria.^29^ One key observation from the present study was that at 4 years of age, the most frequently observed VSS level in our sample was level IV (the most severe) with a predicted marginal probability of 57%. However, by 10 years of age, the most frequently observed VSS level was I or II, with a predicted marginal probability of 51%. These results indicate a clear shift from more to less severe impairment in terms of change in VSS levels with age. The findings from the present study extend earlier work on gains in speech development in children with CP by demonstrating that these changes register on an ordinal scale as measured by VSS levels. Specifically, the present study showed that on average, children’s VSS levels shifted down by approximately one level in the 6-year time course of this study, reflecting improved speech performance and reduced perceived severity of speech involvement. While one VSS level may seem relatively small, the VSS is a blunt measure that comprises only four levels; thus, a one-level change is a 25% shift on this measure. Importantly, none of the children in our sample had a final VSS level that was higher than their first rating, suggesting that the perception of speech severity is not likely to deteriorate over time in children with CP.

The findings for speech from the present study contrast with longitudinal stability research on gross and fine motor classification ratings over time. Specifically, GMFCS and MACS levels are expected to remain consistent over time and are used to offer perspective on the potential for change within, rather than across, levels with respect to expected developmental growth curves.^26,39^ Notably, however, GMFCS levels are based on independence in sitting and walking; MACS ratings are based on holding objects, all of which are milestones reached in typical development by 12 months.^6,7^ Typical speech development is well known to occur throughout childhood. Later-emerging speech sounds are not mastered until 5 to 6 years in English.^40^ Recent work suggested that children in the lower deciles of typical growth curves are still making intelligibility gains as late as 10 years of age.^28^ Thus, the time course of speech development is one that begins and ends later in development than the time course for motor skills captured by the GMFCS and MACS. It is therefore difficult to determine the extent to which VSS ratings in children with CP reflect impairment secondary to CP versus typical speech developmental immaturity or some combination therein. Further study is necessary to determine the age at which VSS levels stabilize, and are thereafter consistent over time, as is the case with the GMFCS and MACS. However, it is clear from the present study that there are different growth expectations for VSS levels in children between the ages of 4 and 10 years than there are for ratings of gross and fine motor ability.

### VSS level at 4 years of age is highly predictive of VSS level at 10 years of age

The second key finding was that despite overall improvement over time, different initial age 4 years VSS levels predicted different VSS outcomes at age 10 years. Not surprisingly, children with initial VSS level I or II ratings had an 98% and 87% chance respectively of being in VSS level I at age 10 years, demonstrating excellent speech prognoses.

Children who were in VSS level III at age 4 years had a 48% chance of being in VSS level I, a 39% chance of being in VSS level II, and a 9% chance of staying at VSS level III at age 10 years. These findings suggest that children rated as being in VSS level III at age 4 years were very likely to show reductions in speech impairment severity, with nearly half reaching the least severe VSS level by age 10 years. VSS levels are meant to reflect speech severity, which is closely related to speech intelligibility; however, studies quantifying the relationship between VSS levels and intelligibility are limited. We do not know from the data in the present study what the range of intelligibility performance was for children in each VSS level, but this information could inform expectations for within-level change over time, as well as define between-level intelligibility thresholds. Ultimately, the findings from the present study suggest that children in VSS level III at age 4 years showed a good prognosis for speech improvement. It is important to note that among children who started in VSS level III or better, across all ages, approximately 43% were receiving some form of speech therapy. This therapy took the form of whatever was provided within the child’s environment and included school-based services for some children and clinic or medically based speech therapy services for other children. Specific data regarding the nature of treatment, intensity of treatment, and progress in treatment are not available; however, it is very likely that these services contributed to growth, at least for some children. Studies that quantify the contribution of treatment to change in speech production are needed to begin to understand the many variables that mediate and moderate speech and language outcomes for children with CP.

Children who were in VSS level IV at age 4 years as a group generally showed less change in their VSS levels over time. Specifically, these children had a 62% chance of being in VSS level IV, a 23% chance of being in VSS level III, and a 13% chance of being in VSS level II at age 10 years. Thus, most children will remain without functional speech capability (VSS level IV) or with very restricted speech ability (VSS level III). The findings for children classified in VSS level IV from the present study show some consistency with research examining GMFCS and MACS ratings over time, namely that the most extreme levels (I and V in both systems) are the most stable compared to mid-classification levels.^11,12^ Again, the specific range of intelligibility values encompassed within each VSS level has the potential to provide important information that refines the findings from the present study and informs the magnitude of within-VSS level change that children may be demonstrating with development. Overall, however, our findings suggest that the prognosis for development of functional speech is very guarded for children who are in VSS level IV at age 4 years. Thus, there is a clear and immediate need for treatment focused on AAC modalities for these children to foster the development of functional expressive communication abilities. There is also a need for tools to identify these children before the age of 4 years, which is the earliest age for use of the VSS.^16^

### Limitations and future directions

This study was the first to examine and comprehensively model longitudinal change in perceived speech severity among children with CP using an ordinal rating scale. These findings provide an important foundation for predicting functional speech outcomes in children with CP. Nevertheless, there were several limitations that must be considered when generalizing the findings from this study.

All participating children were pooled into one model that did not account for speech-specific or other diagnosis factors including CP subtype, speech therapy dosage, spasticity treatments, seizure activity, and epilepsy. We chose to focus only on VSS change without consideration for other covariates because our research goal was to describe how severity ratings changed with age in this sample. Moreover, including CP subtypes or GMFCS ratings would have subdivided each age-by-rating cell and reduced our model’s precision and power. Future studies are necessary to examine differences in longitudinal classification patterns in a larger sample of children that account for other diagnostic factors to elucidate any impact on speech outcomes and speech growth.

Similarly, across our sample of 101 children, we did not have equal representation of children within each VSS level at age 4 years. Specifically, only 5% of children started in VSS level I and 60% of children started in VSS level IV; the extent to which this represents the population is unknown. However, because the data from this study were taken from a longitudinal project focused on speech and language development, there may be self-selection bias of families with children who have significant speech concerns being over-represented in our sample. Future work with population-based samples could address this issue.

In the present study, we observed speech development in children with CP between the ages of 4 and 10 years. We do not know to what extent our findings would follow a similar trajectory for children at earlier or later ages. In future research, we aim to compare longitudinal trends of VSS ratings among a wider age range, spanning throughout early childhood and into young adulthood to observe the long-term impact of speech impairment in CP. The VSS was developed for children aged 4 years and older; however, examination of speech at younger ages is necessary to identify and characterize speech impairment at the earliest possible time. Future work should examine the potential for the classification of speech abilities at even younger ages. There exists a longstanding body of research on typical prelinguistic vocal patterns^41,42^ that could be leveraged for the study of early vocal development in children with CP. There is clear need for future work identifying predictors of speech impairments in infants at risk for CP.

### Implications

The present study indicates that early speech impairment severity classification is highly predictive of performance at older ages in childhood. Our findings directly inform clinicians’ ability to make speech prognoses for children with CP as young as 4 years of age to inform treatment decision-making with regard to AAC needs. Informing treatment directions in speech therapy at the youngest possible age has important potential to improve communication outcomes in children with CP throughout the life span.

## Supplementary Material

Long et al 2022 SuppMat

## Figures and Tables

**TABLE 1:** Summary statistics of children with cerebral palsy at each age

Age group, years	Mean age, months (SD)	Age band, months	*n* at each age	Percentage receiving speech therapy	Percentage not receiving speech therapy	Percentage with speech therapy unknown
4	51.5 (2.8)	48–60	98	62	25	13
6	74.7 (2.1)	71–84	100	73	26	1
8	100.1 (4.4)	96–108^a^	72	64	32	4
10	125.1 (5.3)	120–132^a^	58	64	29	7

Notes:

a The 8- and 10-year age bands did not contain mutually exclusive ages because of limitations with regard to the specific timing of each visit for each child. Specifically, one child with available data at 83 months was included in the age 8 years group because they already had data represented at age 6 years. Similarly, a child with available data at 104 months was included in the age 10 years group because they already had a data point represented in the age 8 years group. The critical variable was that no individual child was represented more than once in a given age level.

**TABLE 2:** Demographic and clinical characteristics of children with CP (*n* = 101)

Demographic/clinical characteristic	
Total number of visits across all children	328

Median number of visits per child	3

Males, *n*	58

Females, *n*	43

Type of CP, *n* (%)	
Spastic	73 (72)
Hypotonic	4 (4)
Ataxic	5 (5)
Dyskinetic	1 (1)
Mixed	5 (5)
Unknown	13 (13)

Ethnicity, *n* (%)	
Asian	1 (1)
Black	4 (4)
White	91 (90)
White and Asian	1 (1)
White and Black	2 (2)
Hispanic	2 (2)

Number of children contributing data, *n* (%)	
Four of four time points	49 (48)
Three of four time points	28 (28)
Two of four time points	24 (24)

GMFCS level, *n* (%)	
I	26 (26)
II	25 (25)
III	5 (5)
IV	20 (19)
V	25 (25)

Abbreviations: CP, cerebral palsy, GMFCS, Gross Motor Function Classification System.

**TABLE 3:** Age 10 years VSS levels (observed and model-estimated) by age 4 years VSS levels

Age 4 years VSS level	Observed age 10 years VSS level						Model-estimated mean rating (95% credible interval)	Model-estimated age 10 years probabilities (95% credible interval)			
	*n*	I	II	III	IV	(Missing)		I	II	III	IV
I	5	5	0	0	0	0	1.0 (1.0–1.2)	0.98 (0.86–1.0)	0.02 (0–0.13)	0 (0–0.02)	0 (0)
II	17	8	2	0	0	7	1.1 (1.0–1.4)	0.87 (0.69–0.97)	0.12 (0.03–0.27)	0.01 (0–0.05)	0 (0–0.02)
III	17	4	8	1	0	4	1.7 (1.4–2.0)	0.48 (0.30–0.67)	0.39 (0.24–0.55)	0.09 (0.03–0.17)	0.03 (0–0.11)
IV	59	1	3	7	17	31	3.5 (3.2–3.7)	0.01 (0–0.06)	0.13 (0.06–0.24)	0.23 (0.13–0.36)	0.62 (0.45–0.77)
(Missing)	3	0	1	1	0	1	–	–	–	–	–

Abbreviation: VSS, Viking Speech Scale.

## Data Availability

The data that support the findings of this study are available on request from the corresponding author. The data are not publicly available due to privacy or ethical restrictions.

## What this paper adds

- Children with early Viking Speech Scale (VSS) ratings below level IV have a good prognosis for speech improvement.
- Children with early VSS level IV ratings are likely to remain at VSS level IV over time.
- Children did not show worsening of VSS level between the ages of 4 and 10 years.

## Abbreviations:

AAC: Augmentative and alternative communication
MACS: Manual Ability Classification System
VSS: Viking Speech Scale
