# Supplementary material for: Longitudinal change in speech classification between 4 and 10 years in children with cerebral palsy
Source: Dev Med Child Neurol. 2022 Mar 9;64(9):1096–105. doi: 10.1111/dmcn.15198 (PMC9339470; doi:10.1111/dmcn.15198)
Supplement: Supplementary file 1 — Appendix S1: Statistical supplementary materials. [file DMCN-64-1096-s001.docx]

Statistical Supplemental Materials for ‘Longitudinal change in speech classification between 4 and 10 years in children with cerebral palsy’

Tristan Mahr

This document describes the statistical analyses used in the manuscript. All of the code shown is for the R programming language (vers. 4.1.1, R Core Team, 2021).

## Data

Our data features repeated measurements of VSS levels rating. The first few rows of our data set would be:

library(tidyverse)
data
#> # A tibble: 328 x 4
#> child age_group age_group_4 vss_rating
#> <chr> <dbl> <dbl> <ord>
#> 1 p001 4 0 4
#> 2 p001 6 2 3
#> 3 p002 4 0 4
#> 4 p002 6 2 3
#> 5 p002 8 4 2
#> 6 p002 10 6 2
#> 7 p003 4 0 2
#> 8 p003 6 2 2
#> 9 p003 10 6 1
#> 10 p004 4 0 4
#> # ... with 318 more rows

unique(data$vss_rating)
#> [1] 4 3 2 1
#> Levels: 1 < 2 < 3 < 4

where child uniquely identifies a child, age_group is the child’s age in years, age_group_4 is the age in years minus 4 (so that the model intercepts are age-4 estimates), and vss_rating is an ordered categorical variable with the levels 1, 2, 3, 4.

## Model for research question 1

We use the brms package (vers. 2.16.1, Bürkner, 2017) to fit a Bayesian ordinal regression model in Stan (vers. 2.27.0, Carpenter et al., 2017). We describe the syntax of the model below.

### Preliminaries

By convention, we need three things for a generalized linear model in R:

- a model family specifying a link function and response distribution
- a model formula specifying response and predictor variables
- data

We already have seen the **data** for the model, so we review the family and formula arguments.

We use the cumulative(link = "logit") **family** to perform ordinal regression by estimating rating thresholds using cumulative probabilities on the log-odds (logit) scale. For more about this ordinal models in brms, see the tutorial in Bürkner & Vuorre (2019). Broadly speaking, we assume there is a latent continuous speech severity scale and the ratings carve up this variable into different regions as in the image below:


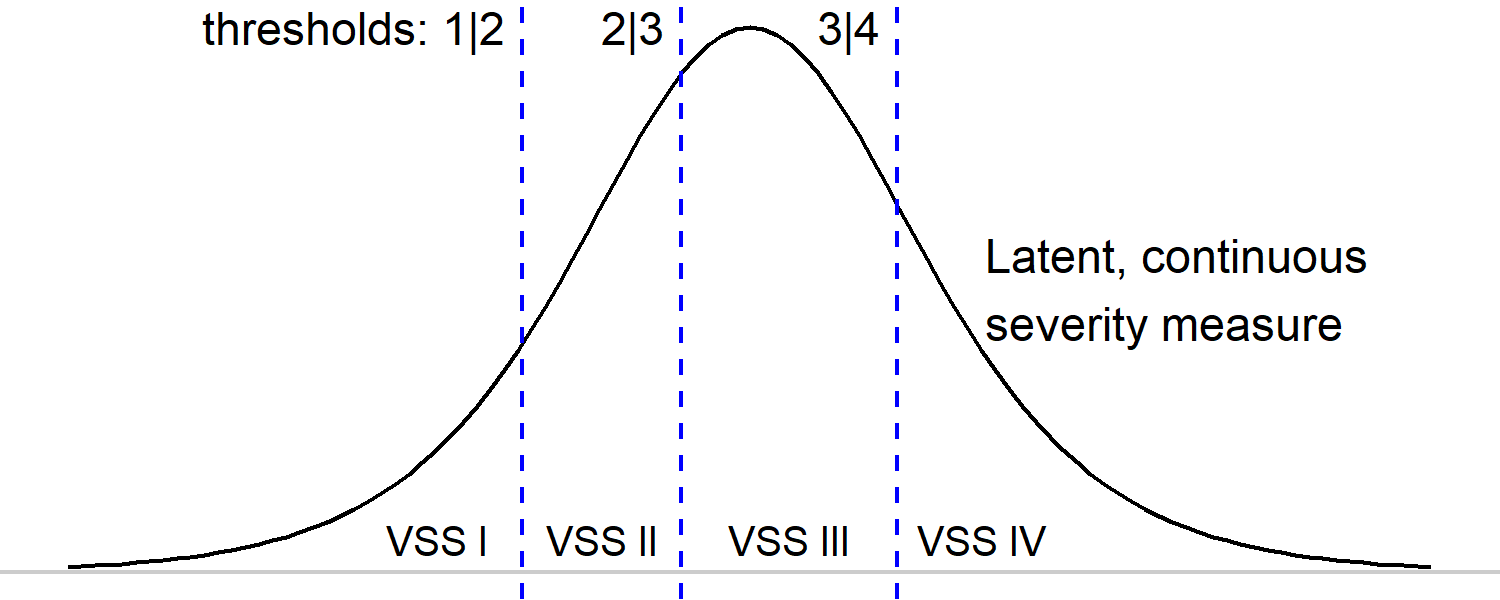


Diagram of model intuition.

Our model’s job is to estimate these thresholds between ratings and how they change with age within children. These thresholds are estimated on the logit (log-odds) scale, so they represent cumulative rating probabilities on the logit scale.

brms uses R’s modeling **formula** syntax in order to specify a mixed effect model. We illustrate the syntax by elaborating from an intercept-only model to our fully specified model:

# estimate three thresholds: 1|2, 2|3, 3|4
vss_rating ~ 1

# estimate thresholds at x = 0 and how they change with age
vss_rating ~ 1 + age_group_4

# estimate thresholds at x = 0 for an average child and how the
# average thresholds change with age. allow each child to have their
# own thresholds by computing an adjustment away from the average
# threshold (by-child random intercepts).
vss_rating ~ 1 + age_group_4 + (1 | child)

# do the same as above but also estimate by-child adjustments for
# age effect (by child random age slopes)
vss_rating ~ 1 + age_group_4 + (1 + age_group_4 | child)

We are using a Bayesian model, so we need one additional thing for our model besides the data, family and formula: **priors** on model parameters. These priors setup plausible values for model parameters before we see any data. The model then updates these prior distributions by using the data to obtain a posterior distribution. We use the default flat and weakly informative priors for our models, so we are *not* ruling out any parameter values based on domain knowledge. In this respect, the prior distributions are computational devices: we use them to get the model machinery going.

Below are these default priors:

library(brms)
priors <- get_prior(
 formula = vss_rating ~ 1 + age_group_4 + (1 + age_group_4 | child),
 family = cumulative(),
 data = data,
)

priors

#> prior class coef group
#> (flat) b
#> (flat) b age_group_4
#> lkj(1) cor
#> lkj(1) cor child
#> student_t(3, 0, 2.5) Intercept
#> student_t(3, 0, 2.5) Intercept 1
#> student_t(3, 0, 2.5) Intercept 2
#> student_t(3, 0, 2.5) Intercept 3
#> student_t(3, 0, 2.5) sd
#> student_t(3, 0, 2.5) sd child
#> student_t(3, 0, 2.5) sd age_group_4 child
#> student_t(3, 0, 2.5) sd Intercept child

Many of these rows are redundant, describing a default prior for a class of parameters (like b for fixed effects) and then the priors for each member of that class (like age_group_4 of class b). In general, we have flat priors on fixed effects terms, wide-tailed Student distributions with three degrees of freedom for the population average rating thresholds (class Intercept) and for the random-effect variances (class sd), and a uniform prior over correlation matrices (lkj(1) for class cor) for the correlation of random effect parameters.

### Model code and output

Below is the actual code use to fit the model. It’s stored in a custom function, There are additional options specified here including random number seed (for reproducibility) and options for how to run the Monte Carlo sampling by Stan:

model <- brm(
 vss_rating ~ 1 + age_group_4 + (1 + age_group_4 | child),
 data = data,
 family = cumulative(),
 backend = "cmdstanr",
 cores = 8,
 chains = 8,
 seed = 20210929,
 control = list(adapt_delta = .9)
)

The model computes runs 2000 sampling iterations on 8 sampling chains. The first 1000 are warm-up iterations where the sampler adapts to the model and they are discarded, leaving us with a posterior distribution of 8,000 draws of model parameters:

nchains(model)
#> [1] 8
niterations(model)
#> [1] 1000
ndraws(model)
#> [1] 8000

The model passes Hamiltonian Monte Carlo diagnostics:

rstan::check_hmc_diagnostics(model$fit)
#>
#> Divergences:
#> 0 of 8000 iterations ended with a divergence.
#>
#> Tree depth:
#> 0 of 8000 iterations saturated the maximum tree depth of 10.
#>
#> Energy:
#> E-BFMI indicated no pathological behavior.

The model parameters have the following estimates:

summary(model, robust = TRUE)
#> Family: cumulative
#> Links: mu = logit; disc = identity
#> Formula: vss_rating ~ age_group_4 + (age_group_4 | child)
#> Data: data (Number of observations: 328)
#> Draws: 8 chains, each with iter = 1000; warmup = 0; thin = 1;
#> total post-warmup draws = 8000
#>
#> Group-Level Effects:
#> ~child (Number of levels: 101)
#> Estimate Est.Error l-95% CI u-95% CI Rhat
#> sd(Intercept) 5.90 1.04 4.21 8.40 1.00
#> sd(age_group_4) 0.49 0.22 0.10 0.97 1.01
#> cor(Intercept,age_group_4) 0.65 0.27 -0.08 0.98 1.00
#> Bulk_ESS Tail_ESS
#> sd(Intercept) 1325 2682
#> sd(age_group_4) 1105 1594
#> cor(Intercept,age_group_4) 1941 2386
#>
#> Population-Level Effects:
#> Estimate Est.Error l-95% CI u-95% CI Rhat Bulk_ESS
#> Intercept[1] -10.57 1.45 -13.98 -8.14 1.00 1461
#> Intercept[2] -4.98 0.98 -7.20 -3.33 1.00 1372
#> Intercept[3] -1.01 0.74 -2.64 0.36 1.00 1468
#> age_group_4 -0.87 0.16 -1.22 -0.58 1.00 2603
#> Tail_ESS
#> Intercept[1] 2435
#> Intercept[2] 2537
#> Intercept[3] 2891
#> age_group_4 3288
#>
#> Family Specific Parameters:
#> Estimate Est.Error l-95% CI u-95% CI Rhat Bulk_ESS Tail_ESS
#> disc 1.00 0.00 1.00 1.00 NA NA NA
#>
#> Draws were sampled using sample(hmc). For each parameter, Bulk_ESS
#> and Tail_ESS are effective sample size measures, and Rhat is the potential
#> scale reduction factor on split chains (at convergence, Rhat = 1).

Group-Level Effects describes the random effects variances and correlations. Population-Level effects describe the rating thresholds for an average child at age 4 with the Intercept[] terms and the age slope for an average child age_group_4. Family Specific Parameters is not relevant here because we hard code the shape of the latent severity scale.

Within each of these, we have summary and diagnostic statistics for model parameters:

- Estimate is the median of the posterior distribution
- Est.Error is the median absolute deviation of the posterior distribution
- l-95% CI, u-95% CI provide the 95% posterior interval
- Rhat, Bulk_ESS, Tail_ESS are diagnostic measures described in the output. They all have acceptable values.

Exponentiating -2 times the age coefficient gives the odds ratio reported in the manuscript for a two-year-increase in age on rating probabilities:

b_age <- fixef(model, robust = TRUE)["age_group_4", ]
exp(-2 * b_age) |> round(1)
#> Estimate Est.Error Q2.5 Q97.5
#> 5.7 0.7 11.6 3.2

Posterior expectations from the model are the expected probability of each VSS rating level. We can get predictions for the model’s average child by creating a “fake” child and asking for the prediction but not conditioning on the child variable (re_formula = NA).

library(tidybayes)
#>
#> Attaching package: 'tidybayes'
#> The following objects are masked from 'package:brms':
#>
#> dstudent_t, pstudent_t, qstudent_t, rstudent_t

one_new_child <- data.frame(
 child = "fake",
 age_group_4 = c(0, 2, 4, 6)
)

draws_average_child <- one_new_child %>%
 add_epred_draws(
 model,
 allow_new_levels = TRUE,
 re_formula = NA
 )

We have 8000 posterior samples for each age and rating level:

count(draws_average_child, child, age_group_4, .category)
#> # A tibble: 16 x 5
#> # Groups: child, age_group_4, .row, .category [16]
#> child age_group_4 .row .category n
#> <chr> <dbl> <int> <fct> <int>
#> 1 fake 0 1 1 8000
#> 2 fake 0 1 2 8000
#> 3 fake 0 1 3 8000
#> 4 fake 0 1 4 8000
#> 5 fake 2 2 1 8000
#> 6 fake 2 2 2 8000
#> 7 fake 2 2 3 8000
#> 8 fake 2 2 4 8000
#> 9 fake 4 3 1 8000
#> 10 fake 4 3 2 8000
#> 11 fake 4 3 3 8000
#> 12 fake 4 3 4 8000
#> 13 fake 6 4 1 8000
#> 14 fake 6 4 2 8000
#> 15 fake 6 4 3 8000
#> 16 fake 6 4 4 8000

We can do two things now: Compute quantiles on the probabilities or compute expected ratings.

draws_average_child %>%
 ggdist::median_qi() %>%
 select(child:.upper)
#> # A tibble: 16 x 7
#> child age_group_4 .row .category .epred .lower .upper
#> <chr> <dbl> <int> <fct> <dbl> <dbl> <dbl>
#> 1 fake 0 1 1 0.0000256 0.000000848 0.000292
#> 2 fake 0 1 2 0.00679 0.000743 0.0342
#> 3 fake 0 1 3 0.259 0.0649 0.564
#> 4 fake 0 1 4 0.734 0.411 0.934
#> 5 fake 2 2 1 0.000147 0.00000676 0.00129
#> 6 fake 2 2 2 0.0374 0.00538 0.151
#> 7 fake 2 2 3 0.622 0.300 0.818
#> 8 fake 2 2 4 0.329 0.102 0.688
#> 9 fake 4 3 1 0.000838 0.0000492 0.00616
#> 10 fake 4 3 2 0.181 0.0320 0.511
#> 11 fake 4 3 3 0.704 0.449 0.849
#> 12 fake 4 3 4 0.0806 0.0134 0.311
#> 13 fake 6 4 1 0.00481 0.000304 0.0332
#> 14 fake 6 4 2 0.551 0.141 0.873
#> 15 fake 6 4 3 0.425 0.103 0.774
#> 16 fake 6 4 4 0.0151 0.00141 0.104

The .epred column is the posterior median probability for each rating level (.category) and .lower and .upper are 95% quantiles. The probabilities shift from favoring level 4 at age 4 to favoring level 2 at age 10.

If we multiple each rating’s numerical level by its probability and sum the results, we get an average or expected rating.

draws_expected_ratings <- draws_average_child %>%
 ungroup() %>%
 mutate(
 weight = as.numeric(.category) * .epred
 ) %>%
 group_by(child, age_group_4, .draw) %>%
 summarise(
 expected_rating = sum(weight),
 .groups = "drop"
 )
draws_expected_ratings
#> # A tibble: 32,000 x 4
#> child age_group_4 .draw expected_rating
#> <chr> <dbl> <int> <dbl>
#> 1 fake 0 1 3.67
#> 2 fake 0 2 3.76
#> 3 fake 0 3 3.62
#> 4 fake 0 4 3.84
#> 5 fake 0 5 3.82
#> 6 fake 0 6 3.73
#> 7 fake 0 7 3.75
#> 8 fake 0 8 3.55
#> 9 fake 0 9 3.53
#> 10 fake 0 10 3.59
#> # ... with 31,990 more rows

Then compute quantiles on these expected ratings:

draws_expected_ratings %>%
 group_by(child, age_group_4) %>%
 ggdist::median_qi(expected_rating)
#> # A tibble: 4 x 8
#> child age_group_4 expected_rating .lower .upper .width .point .interval
#> <chr> <dbl> <dbl> <dbl> <dbl> <dbl> <chr> <chr>
#> 1 fake 0 3.73 3.39 3.93 0.95 median qi
#> 2 fake 2 3.29 2.98 3.68 0.95 median qi
#> 3 fake 4 2.90 2.50 3.26 0.95 median qi
#> 4 fake 6 2.45 2.09 2.95 0.95 median qi

But the problem with these estimates so far are they are conditional expectations. They are the expectations for an average child, a child whose random effect adjustments have been zeroed out. They do not describe an average over children. We need to compute a *marginal mean*.

### Computation of marginal means

The model’s fixed effects estimates ignore between-child variability in thresholds and age-slopes so when we compute the (marginal) average rating probabilities, we’d need average over this variability. We used the following procedure.

1. Create 100 new unobserved children.
2. Generate posterior expectations for these children.
3. For each posterior draw, average these 100 children together.
4. We now have a posterior distribution of 8,000 means that average over the variability in 100 children.

Here are the new chidren:

data_100_new_kids
#> # A tibble: 400 x 3
#> child age_group_4 age_group
#> <chr> <dbl> <dbl>
#> 1 .c001 0 4
#> 2 .c001 2 6
#> 3 .c001 4 8
#> 4 .c001 6 10
#> 5 .c002 0 4
#> 6 .c002 2 6
#> 7 .c002 4 8
#> 8 .c002 6 10
#> 9 .c003 0 4
#> 10 .c003 2 6
#> # ... with 390 more rows

Then we compute expectations for them:

draws_posterior_epred_100_new_children <- data_100_new_kids %>%
 tidybayes::add_epred_draws(
 model,
 # include all random effects
 re_formula = NULL,
 allow_new_levels = TRUE,
 sample_new_levels = "gaussian"
 )

And average the children within each draw together:

draws_posterior_epred_100_new_children_means <-
 draws_posterior_epred_100_new_children %>%
 group_by(.draw, age_group, age_group_4, .category) %>%
 summarise(
 .epred = mean(.epred),
 .groups = "drop"
 )
draws_posterior_epred_100_new_children_means

draws_posterior_epred_100_new_children_means
#> # A tibble: 128,000 x 5
#> .draw age_group_4 age_group .category .epred
#> <int> <dbl> <dbl> <fct> <dbl>
#> 1 1 0 4 1 0.0417
#> 2 1 0 4 2 0.138
#> 3 1 0 4 3 0.282
#> 4 1 0 4 4 0.539
#> 5 1 2 6 1 0.0937
#> 6 1 2 6 2 0.225
#> 7 1 2 6 3 0.260
#> 8 1 2 6 4 0.421
#> 9 1 4 8 1 0.184
#> 10 1 4 8 2 0.278
#> # ... with 127,990 more rows

This code then provides the marginal VSS level rating probabilities reported in the manuscript:

#> # A tibble: 16 x 6
#> age_group age_group_4 .category .epred .lower .upper
#> <dbl> <dbl> <fct> <dbl> <dbl> <dbl>
#> 1 4 0 1 0.0417 0.00791 0.108
#> 2 4 0 2 0.163 0.0977 0.243
#> 3 4 0 3 0.223 0.150 0.310
#> 4 4 0 4 0.565 0.443 0.685
#> 5 6 2 1 0.0952 0.0380 0.180
#> 6 6 2 2 0.216 0.140 0.308
#> 7 6 2 3 0.222 0.153 0.306
#> 8 6 2 4 0.457 0.338 0.579
#> 9 8 4 1 0.171 0.0916 0.274
#> 10 8 4 2 0.245 0.166 0.338
#> 11 8 4 3 0.206 0.138 0.288
#> 12 8 4 4 0.372 0.256 0.493
#> 13 10 6 1 0.257 0.158 0.374
#> 14 10 6 2 0.250 0.167 0.344
#> 15 10 6 3 0.181 0.116 0.262
#> 16 10 6 4 0.304 0.190 0.437

Marginal expected ratings were computed by taking expected ratings from the average probabilities within each draw.

### Child-specific age trajectories

These are not reported in the manuscript, but our model did estimate growth trajectories for each child. We can visualize these estimates with observed data. We can see what the model learned/predicted for children with partial data (less than four observations).

First note that there are 4 possible ratings at 4 timepoints so there are many duplicated patterns of data of data. Here are the most frequent patterns:

data_vss_wide %>%
 count(
 vss_at_4,
 vss_at_6,
 vss_at_8,
 vss_at_10,
 name = "num_children"
 ) %>%
 arrange(desc(num_children))

The 14 children with all 4’s have the same estimated trajectory so we only need to visualize their data once. The leads to the following plot with one panel per pattern:


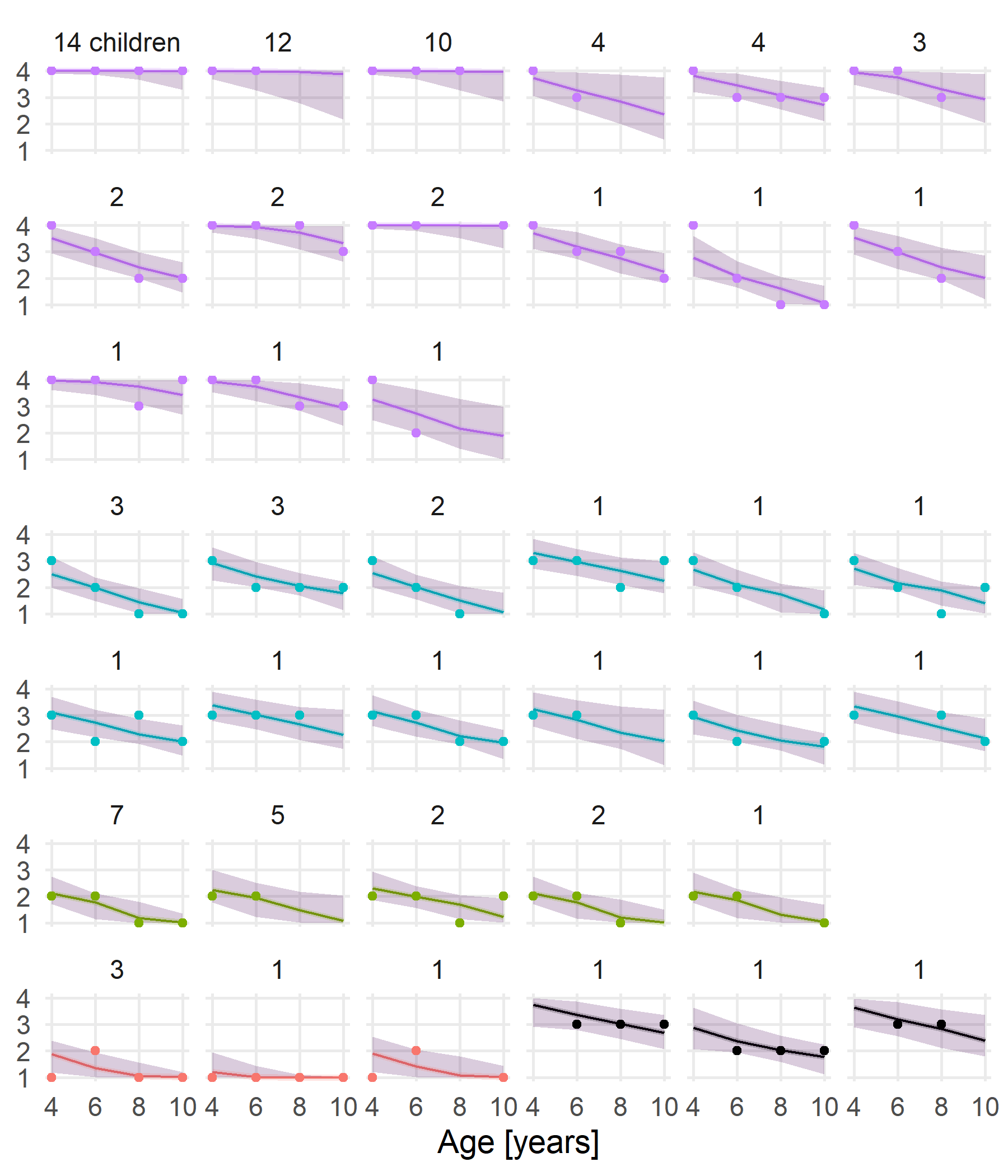


Child-specific trajectories. Points represent observed ratings. Bands are 95% posterior intervals for the expected rating. The numbers over the panels are the number of the children with this pattern of data. Colors represent age-4 rating levels.

## Research question 2 model

For research question 2, we include the initial (age-4) ratings as a categorical predictor variable called starting_rating. We also provide continuous version of it called starting_rating_cont. We use the continuous version when compute the age by initial rating interaction.

data2 %>%
 distinct(starting_rating, starting_rating_cont)
#> # A tibble: 4 x 2
#> starting_rating starting_rating_cont
#> <fct> <dbl>
#> 1 4 3
#> 2 2 1
#> 3 3 2
#> 4 1 0

For this model, we centered age so that age 10 years occurs at 0 (the model intercept), using the variable age_group_10. The data has the following structure:

data2
#> # A tibble: 222 x 5
#> child vss_rating starting_rating age_group_10 starting_rating_cont
#> <chr> <ord> <fct> <dbl> <dbl>
#> 1 p001 3 4 -4 3
#> 2 p002 3 4 -4 3
#> 3 p002 2 4 -2 3
#> 4 p002 2 4 0 3
#> 5 p003 2 2 -4 1
#> 6 p003 1 2 0 1
#> 7 p004 4 4 -4 3
#> 8 p004 4 4 -2 3
#> 9 p004 4 4 0 3
#> 10 p005 2 2 -4 1
#> # ... with 212 more rows

### Model code and output

Our model estimates the age trajectories from ages 6 to 8 to 10 by using age-4 VSS level rating as a predictor. Thresholds and age-slopes are adjusted using these starting rating values by using the starting_rating predictor and the age_group_10:starting_rating_cont interaction.

model_t <- brm(
 vss_rating ~
 1 + age_group_10 + starting_rating +
 age_group_10:starting_rating_cont +
 (1 + age_group_10 | child),
 data = data2,
 family = cumulative(),
 backend = "cmdstanr",
 cores = 8,
 chains = 8,
 seed = 20211014,
 control = list(adapt_delta = .95)
)

The model passes model diagnostics:

rstan::check_hmc_diagnostics(model_t$fit)
#>
#> Divergences:
#> 0 of 8000 iterations ended with a divergence.
#>
#> Tree depth:
#> 0 of 8000 iterations saturated the maximum tree depth of 10.
#>
#> Energy:
#> E-BFMI indicated no pathological behavior.

The model has the following output, although our inferences are driven solely by estimated marginal means:

summary(model_t, robust = TRUE)
#> Family: cumulative
#> Links: mu = logit; disc = identity
#> Formula: vss_rating ~ age_group_10 + starting_rating + age_group_10:starting_rating_cont + (age_group_10 | child)
#> Data: data2 (Number of observations: 222)
#> Draws: 8 chains, each with iter = 1000; warmup = 0; thin = 1;
#> total post-warmup draws = 8000
#>
#> Group-Level Effects:
#> ~child (Number of levels: 98)
#> Estimate Est.Error l-95% CI u-95% CI Rhat
#> sd(Intercept) 6.57 1.61 4.07 10.55 1.00
#> sd(age_group_10) 0.99 0.35 0.41 1.87 1.00
#> cor(Intercept,age_group_10) 0.97 0.03 0.78 1.00 1.00
#> Bulk_ESS Tail_ESS
#> sd(Intercept) 1847 2904
#> sd(age_group_10) 2071 3070
#> cor(Intercept,age_group_10) 3469 5136
#>
#> Population-Level Effects:
#> Estimate Est.Error l-95% CI u-95% CI
#> Intercept[1] 14.05 4.36 6.76 24.95
#> Intercept[2] 22.29 5.16 13.71 35.19
#> Intercept[3] 27.36 5.58 18.00 41.20
#> age_group_10 -4.02 1.07 -6.70 -2.34
#> starting_rating2 6.45 2.83 1.44 12.65
#> starting_rating3 14.38 4.22 7.22 24.51
#> starting_rating4 29.37 6.22 19.10 45.15
#> age_group_10:starting_rating_cont 1.32 0.42 0.65 2.37
#> Rhat Bulk_ESS Tail_ESS
#> Intercept[1] 1.00 2553 2963
#> Intercept[2] 1.00 2215 2742
#> Intercept[3] 1.00 2088 2722
#> age_group_10 1.00 2397 2900
#> starting_rating2 1.00 3687 4017
#> starting_rating3 1.00 2484 2946
#> starting_rating4 1.00 1984 2788
#> age_group_10:starting_rating_cont 1.00 2399 3142
#>
#> Family Specific Parameters:
#> Estimate Est.Error l-95% CI u-95% CI Rhat Bulk_ESS Tail_ESS
#> disc 1.00 0.00 1.00 1.00 NA NA NA
#>
#> Draws were sampled using sample(hmc). For each parameter, Bulk_ESS
#> and Tail_ESS are effective sample size measures, and Rhat is the potential
#> scale reduction factor on split chains (at convergence, Rhat = 1).

### Marginal means

We made inferences using marginal means using the same procedure as above. The only adjustment is that we simulate children for each starting (age-4) rating.

1. Simulate 400 new children (100 per initial starting rating).
2. Compute posterior expectations for these children to get rating probabilities.
3. For each posterior draw, compute the average rating probability for each batch of 100 children per initial rating levels.
4. Keep just the age-10 probabilities.
5. We now have a posterior distribution of average age-10 outcomes (rating probabilities) for each age-4 rating level.

## References

Bürkner, P.-C. (2017). brms: An R package for Bayesian multilevel models using Stan. *Journal of Statistical Software*, *80*(1), 1–28. doi:[10.18637/jss.v080.i01](https://doi.org/10.18637/jss.v080.i01)

Bürkner, P.-C., & Vuorre, M. (2019). Ordinal regression models in psychology: A tutorial. *Advances in Methods and Practices in Psychological Science*, *2*(1), 77–101. doi:[10.1177/2515245918823199](https://doi.org/10.1177/2515245918823199)

Carpenter, B., Gelman, A., Hoffman, M., Lee, D., Goodrich, B., Betancourt, M., … Allen Riddell, and. (2017). Stan: A probabilistic programming language. *Journal of Statistical Software*, *76*(1), 1–32. doi:[10.18637/jss.v076.i01](https://doi.org/10.18637/jss.v076.i01)

R Core Team. (2021). *R: A language and environment for statistical computing*. Vienna, Austria: R Foundation for Statistical Computing. Retrieved from <https://www.R-project.org/>
